# Reform in Anatomical Nomenclature

**Published:** 1890-06

**Authors:** 


### REFORM IN ANATOMICAL NOMENCLATURE.

THE following report of the Committee on Anatomical Nomenclature was adopted December 28, 1889, by the Association of American Anatomists without dissent.

The committee recommends:

1. That the adjectives dorsal and ventral be employed in place of *posterior* and *anterior* as commonly used in human anatomy, and in place of *upper* and *lower* as sometimes used in comparative anatomy.

2. That the cornua of the spinal cord, and the spinal nerve-roots, be designated as dorsal and ventral rather than as *posterior* and *anterior*.

3. That the costiferous vertebra be called thoracic rather than *dorsal*.

4. That the *hippocampus minor* be called calcar; the *hippocampus major*, hippocampus; the *pons Varolii*, pons; the *insula Reilii*, insula; *pia mater* and *dura mater*, respectively pia and dura."

Signed by all the members.

JOSEPH LEIDY, *Chairman.*
HARRISON ALLEN,
FRANK BAKER,
THOMAS B. STOWELL,
BURT G. WILDER.

Thomas Dwight was added to the committee.

The following is the report of the Committee on Anatomical Nomenclature of the American Association for the Advancement of Science, with special reference to the brain:

During the past year, some of the members of the Committee have given to the subject intrusted to them as much time as their regular duties would permit. They agree upon one point, viz., the advantages, other things being equal, of *mononyms* (single word terms) over *polyonyms* (terms consisting of two or more words). Before making specific recommendations or presenting a final report, the Committee think it advisable that they and other anatomists should have an opportunity of discussing at leisure the simplified nomenclature which they are informed is employed in certain treatises which will be published during the coming Winter. They therefore ask to be continued.

BURT G. WILDER, *Chairman.*
HARRISON ALLEN,
FRANK BAKER,
HENRY F. OSBORN,
T. B. STOWELL,
*Committee.*

*Note by the Chairman*—The treatises referred to in the above report are Leidy's Human Anatomy, and the following articles in Wood's Reference Handbook of the Medical Sciences, Vol. viii.: By E. C. Spitzka, Spinal Cord and Histology of the Brain; W. Browning, Vessels of the Brain; S. H. Gage and B. G. Wilder, Anatomical Terminology; B. G. Wilder, Anatomy of the Brain, Malformations of the Brain, and Methods of Dissection, etc.

After careful consideration of what had been written by Barclay, Owen, and Pye-Smith, on the 29th of August, 1880, at the Boston meeting of the American Association for the Advancement of Science, Professor Wilder, of Cornell University, made the first systematic effort in this country to simplify anatomical nomenclature, especially with reference to the brain. This paper was briefly reported in the New York *Medical Record*, September 18, 1880. Since that time he has published numerous papers, bearing more or less directly upon this subject, and this system is embodied in "Anatomical Technology," by Wilder and Gage, and in the article "Gross Anatomy of the Brain," in Vol. viii., of the Reference Handbook of Medical Sciences.

In 1884, at Prof. Wilder's suggestion, committees on nomenclature were appointed by the American Neurological Association, and the American Association for Advancement of Science. It is to be hoped that the former may present a preliminary report at its next meeting, to be held in Philadelphia, June 4-6, 1890. The report of the latter has appeared, and favors the adoption of mononyms, one word terms (see above). They have also asked further time for discussing the simplified nomenclature, and it is to be hoped they will also favor the adoption of the system as proposed by Dr. Wilder in 1880.

The committee of the Association of American Anatomists (as will be seen by referring to their report printed above) recommended the terms "Dorsal" and "Ventral," in place of the less exact terms, upper and lower, or anterior and posterior, and also the employment of several mononyms in place of the polyonyms heretofore in vogue.

It is worthy of note that both the principle of mononyms, and the specific terms adopted by these committees, formed prominent features of Prof. Wilder's original paper in 1880. Since that time he has imbued the necessity of reform into not only his students at Cornell University, but also in the masses of scientific men, eager for the advancement of their science.

Slowly and gradually, as all reforms advance, this one has been steadily gaining ground, and although misinterpreted and attacked, Prof. Wilder has not lost faith, and has preserved his equanimity with surprising calmness (see New York *Medical Record*, October 4, 1886), against erroneous and rather spiteful editorials as have appeared in some of the metropolitan journals.

It is gratifying to see some, who at first have been adverse to the new system, now indorse and use it, and others acting through committees favoring its adoption. Those of us who had the pleasure of listening to Dr. Wilder's address before the Alumni Association of the Niagara Medical College, can attest with what force and precision parts of the brain were described by means of some of the terms of the proposed nomenclature. The ambiguity and circumlocution of the old terms became patent when contrasted with such terms as meson, cephalic-candad, ental-ectal, dorsal-ventral, peripheral-central, dextral-sinistral, etc. Each of these terms permits of adjective and adverbal terminations, as cephalad, towards the head, etc. The description and location of anatomical tissues and centers is rendered comparatively easy, to say nothing of the accuracy and precision necessary for a perfect comprehension of the subject. We hope that ere long the system may be adopted, not only by the teachers of the various schools, but also by the physician in reporting his cases for the medical journals. In conclusion we reproduce the following paragraph from the article Anatomical Terminology, (Reference Handbook, Vol. viii., p. 522, § 45,) as a compact statement of the reasons for giving his views careful consideration :

> The writer asks a careful consideration of his plans for terminological improvement, because they are based upon an unbiased study of nearly all previous publications; because—as he has reason to think—during the last ten years he has given to the subject more time than has been given by any other anatomist, living or dead; because the linguistic principles involved have been approved by philological authorities; because the actual disturbance of the existing order of things is kept at the minimum; and because the practical availability of the terms has been demonstrated with hundreds of students in both a university and a medical school.
>
> As contrasted with the vocabulary in common use, (at least up to 1880,) that which seems to the writer best calculated to facilitate the advancement and dissemination of accurate anatomical knowledge would consist of terms, which are :

Designatory rather than descriptive.
Impersonal rather than personal (eponymit).
Correlate rather than irrelate.
Conservative rather than eradicating.
Idionymic rather than homonymic.
Coördinate rather than incoördinate.
Classical rather than vernacular.
Dissyllabic or trisyllabic rather than monosyllabic or polysyllabic.
Mononymic rather than polyonymic.
Monoglot rather than polyglot.
Paronymic rather than heteronymic.
Constant rather than varied.

---

General Green B. Raum deserves credit for his attempt to place the pension examination service on a systematic basis. A bill recently introduced at his instance provides for the entering wedge of a system of selecting pension surgeons similar to that adopted by army, navy, and marine hospital service.—*Medical Standard, April.*

